# Species-specific effects of post-wheat green manure incorporation on spring wheat yield and soil greenhouse gas emissions in an arid irrigated system

**DOI:** 10.1371/journal.pone.0360932

**Published:** 2026-10-14

**Authors:** Bing Li, Li Wang, Cundong Li, Zhanyuan Lu, Li Zhang, Hongbo Liu, Juan Li, Dejian Zhang

**Affiliations:** 1 School of Life Sciences, Inner Mongolia University/Key Laboratory of Biology of Forage and Special Crops, Ministry of Education, Hohhot, Inner Mongolia, China; 2 College of Agronomy, Hebei Agricultural University, Baoding, Hebei, China; 3 Inner Mongolia Academy of Agricultural and Animal Husbandry Sciences, Hohhot, Inner Mongolia, China; 4 Hohhot Agricultural and Animal Husbandry Technology Extension Center, Hohhot, Inner Mongolia, China; ICAR-National Rice Research Institute, INDIA

## Abstract

Post-wheat green manure incorporation can improve fallow-season resource use and crop productivity, but species-specific effects on soil greenhouse gas emissions and subsequent wheat yield remain unclear in spring wheat systems. A field experiment was conducted from 2022 to 2023 in the Yellow River Basin of Inner Mongolia, China, with five green manure species, namely common vetch, hairy vetch, sesbania, forage rapeseed, and forage oat, and a fallow control. Soil CO_2_, CH_4_, and N_2_O emissions, global warming potential (GWP), greenhouse gas intensity (GHGI), wheat growth, and grain yield were evaluated. The system acted as a source of CO_2_ and N_2_O and a sink for CH_4_. Compared with fallow, green manure incorporation numerically increased cumulative CO2 emissions by 7.29%−44.30% and GWP by 7.44%−39.64%, although treatment differences in GWP were not significant, with CO2 contributing more than 85% of total GWP. It also increased grain yield by 2.96%−11.95%. Despite the numerical increase in GWP, GHGI differed among species. Hairy vetch achieved the highest yield and lowest GHGI, common vetch reduced GHGI relative to fallow, and forage oat showed high yield with the lowest cumulative N_2_O emission. These results suggest that hairy vetch, common vetch, and forage oat are suitable for post-wheat incorporation in this region.

## Introduction

Agricultural production, while ensuring food supply, is also a significant source of greenhouse gas emissions from farmland [1]. The emission or absorption processes of CO_2_, CH_4_ and N_2_O in farmland soil are jointly regulated by factors such as crop planting systems, nitrogen input, crop residue input and soil water and heat conditions [2]. In the context of low-carbon agricultural development, the evaluation of agricultural management measures cannot be based solely on grain yield [3]. It is also necessary to take into account the greenhouse effect per unit area and the greenhouse gas emission intensity per unit yield. The global warming potential (GWP) can comprehensively represent the warming effects of CO_2_, CH_4_ and N_2_O on a unit area scale, while the greenhouse gas emission intensity (GHGI) links greenhouse gas emissions with crop yields and can be used to evaluate the relationship between yield formation and greenhouse gas emission intensity [4,5]. Therefore, in the spring wheat cultivation system, how to increase yield while controlling GWP and reducing GHGI is the core issue that needs to be addressed for optimizing the low-carbon and high-yield cultivation system.

Post-wheat green manure incorporation is an important way to enhance the utilization efficiency of resources during the off-season and improve the productivity of the following wheat crops [6]. After the spring wheat harvest in the Yellow River Basin of Inner Mongolia, there are still certain amounts of light, heat, water and land resources available. However, the traditional fallow system is unable to fully utilize these resources [7]. Green manure crops can accumulate biomass after the harvest of wheat [8]. After being plowed back into the soil, they input organic carbon and nitrogen into the soil, thereby influencing the nutritional growth, above-ground dry matter accumulation and yield formation of the following wheat crop [9]. Previous studies have shown that cover crops or legumes can affect the yield of subsequent crops by increasing organic matter input and regulating nitrogen supply [10]. However, this effect is constrained by the type of legumes, the quality of residues, and the process of nutrient release [11]. Therefore, different types of green manure may have different effects on wheat plant height, leaf area index, dry matter accumulation, yield components and grain yield.

The impact of green manure application on soil greenhouse gas emissions is also uncertain [12]. The input of green manure residues provides substrates for soil carbon and nitrogen transformation, which may promote the release of soil CO_2_, and alter N_2_O emissions by influencing the nitrogen transformation process [13]. Meanwhile, the CH_4_ flux in dryland soil is mainly influenced by the relative intensities of methane production and methane oxidation [14]. Changes in soil moisture, aeration conditions, and nitrogen supply during the decomposition of green manure residues may all affect the CH_4_ absorption process. Due to the differences in biomass, C/N ratio of residues, nitrogen supply, and decomposition characteristics among various green manure crops, the direction and intensity of their impacts on CO_2_, CH_4_ and N_2_O may vary [15]. Therefore, green manure incorporation into the soil cannot simply be defined as an emission increase or emission reduction measure. Its production and greenhouse gas effect should be comprehensively evaluated by considering CO_2_, CH_4_, N_2_O, GWP, GHGI and wheat yield [16].

Previous studies have mainly focused on the effects of green manure application on crop yield, nitrogen supply, or single greenhouse gas emissions [17]. However, in the spring wheat-after-cropping system, there are still insufficient studies that simultaneously compare the impacts of different green manure types on soil CO_2_, CH_4_, N_2_O emissions, GWP, GHGI, and wheat yield formation. Especially in the Yellow River basin of Inner Mongolia, there is a lack of systematic evidence regarding whether different green manure crops will lead to differential responses in terms of greenhouse gas emissions and yield formation after being returned to the soil, and whether the increase in yield can reduce the greenhouse gas emission intensity per unit yield. To address this knowledge gap, this study compared five post-wheat green manure species, namely common vetch, hairy vetch, sesbania, forage rapeseed, and forage oat, with a fallow control. Specifically, this study aimed to address two scientific questions: (1) how different post-wheat green manure species affect soil CO_2_, CH_4_ and N_2_O emissions during the subsequent spring wheat season, and thereby alter GWP and GHGI; and (2) whether different green manure species can coordinate yield improvement with reduced yield-scaled greenhouse gas emissions by promoting wheat growth and yield formation. By addressing these questions, this study aimed to identify green manure species suitable for post-wheat incorporation in low-carbon and high-yield spring wheat production systems in the Yellow River Basin of Inner Mongolia.

## Materials and methods

### Experimental site description

The field experiment was conducted from March 2022 to October 2023 at the Xihai Xin Experimental Site in Shulinzhao Town, Dalad Banner, Ordos City, Inner Mongolia Autonomous Region, China (40°29′2″N, 109°52′25″E). The region has a temperate continental monsoon climate, characterized by abundant sunlight, distinct seasonal variation, and a frost-free period of 135–150 days. According to the meteorological records from the Ordos Meteorological Station, the mean annual evaporation ranges from 1950 to 2200 mm, whereas the mean annual precipitation is approximately 240–360 mm, mainly occurring from June to September. The coincidence of rainfall and heat during the growing season makes this area a typical inland arid irrigated agricultural region, where single cropping is generally reliable. However, after wheat harvest, the remaining growing season is relatively short and the accumulated temperature is insufficient to ensure the successful maturation of a second grain crop, thereby limiting double cropping in this region. The dominant crops are wheat and maize. Continuous cropping is common, and conventional tillage is usually practiced after crop harvest. The soil type is mainly fluvo-aquic soil. Before the experiment in 2022, the basic physicochemical properties of the 0–20 cm soil layer were as follows: pH 8.32, soil organic carbon 8.26 g/kg, total nitrogen 0.67 g/kg, available phosphorus 6.39 mg/kg, and available potassium 116.89 mg/kg. The experimental field was flat and had relatively uniform soil fertility. Permission to conduct the field experiment was obtained from the Xihai Xin Experimental Site. The field study was not conducted in a protected area and did not involve protected species.

### Experimental arrangement and field management

The field experiment was initiated in August 2022 after the harvest of the preceding spring wheat crop in mid-July 2022. Different green manure crops were subsequently planted to evaluate their effects on the growth, yield formation, and greenhouse gas emissions of the following wheat crop. The experiment included five green manure treatments and one fallow treatment. Each treatment was replicated three times, resulting in a total of 18 plots arranged in a randomized block design. Each plot covered an area of 144 m^2^, with dimensions of 4.5 m × 32 m. Buffer strips of 0.8 m were established on the northern and southern sides of the experimental field, and 1.0 m buffer strips were established on the eastern and western sides to minimize edge effects. Green manure crops were sown on 3 August 2022. The tested green manure species included common vetch (*Vicia sativa* L), hairy vetch (*Vicia villosa* Roth), sesbania (*Sesbania cannabina* (Retz.) Poir), forage rapeseed (*Brassica napus* L), and forage oat (*Avena sativa* L). Common vetch, hairy vetch, and sesbania are leguminous species, whereas forage rapeseed and forage oat are non-leguminous species. Common vetch, hairy vetch, and sesbania were planted in rows with a row spacing of 30 cm, whereas forage rapeseed and forage oat were manually broadcast. No fertilizer was applied during the green manure growth period to avoid interference from external nutrient inputs. In early October 2022, the green manure biomass was chopped and incorporated into the soil. The chopped residues were 10−15 cm in length, and the incorporation depth was 20−30 cm. After incorporation, winter irrigation was applied uniformly to all plots to promote contact between green manure residues and the plough layer and to improve soil moisture conditions for the subsequent wheat season. The following wheat crop cultivar was Ningchun 4, which was sown on 13 April 2023 at a seeding rate of 450 kg ha^-1^ using a wheat row seeder with a row spacing of 15 cm. A compound fertilizer was applied as basal fertilizer at sowing at a rate of 525 kg ha^-1^, with a nutrient ratio of N-P_2_O_5_-K_2_O = 15-39-8. During the wheat growing season, urea containing ≥46.0% N was manually top-dressed on 12 May and 7 June 2023. The corresponding pure N application rates were 82.8 and 34.5 kg ha^-1^, respectively. During the subsequent wheat growing season, all treatments, including the fallow treatment, received the same basal fertilizer and urea top-dressing rates, irrigation, and other field management practices. Wheat was harvested in mid-July 2023.

### Determination of greenhouse gas emission

Soil nitrous oxide (N_2_O), carbon dioxide (CO_2_), and methane (CH_4_) fluxes were measured using a closed static chamber technique. The chamber system consisted of a removable sampling chamber and a fixed base. The sampling chamber was made of polyethylene and had dimensions of 20 cm × 20 cm × 30 cm. The fixed base was installed at a permanent sampling position in each plot throughout the sampling period, with 3 cm inserted into the soil and 8 cm remaining above the soil surface. During gas sampling, the chamber enclosed the soil surface and root-zone soil within the fixed base. A gas sampling tube was attached to the side wall of the chamber and connected to a three-way valve, which allowed gas collection using a syringe. The chamber bases were installed in each plot one day before the first gas sampling to minimize disturbance caused by base placement. During the seedling stage, wheat seedlings within the chamber area were removed to minimize the influence of aboveground plant tissues on greenhouse gas measurements. During sampling, a KT300 thermometer was inserted into the soil at a depth of 5 cm within the fixed base to record soil temperature. The removable chamber was then placed into the groove of the fixed base and sealed with water to maintain airtight conditions. Gas samples were collected at 10, 20, and 30 min after chamber closure. At each sampling time, a 50 mL syringe was connected to the chamber through the three-way valve. Before gas collection, the syringe plunger was pushed and pulled 5–8 times to ensure sufficient mixing of the air inside the chamber. Subsequently, 40 mL of gas was collected and transferred into an evacuated vial. Gas sampling was conducted between 09:00 and 11:00 to reduce the influence of diurnal variation on flux estimation. After wheat sowing, gas samples were collected on days 1, 3, 5, 7, and 10, and thereafter at 10-day intervals during the wheat growing season. When heavy rainfall or irrigation occurred, sampling was postponed appropriately to avoid disturbance-related bias. The collected gas samples were analyzed using an Agilent 7890A gas chromatograph to determine the concentrations of CO_2_, CH_4_, and N_2_O. CO_2_ was converted to CH_4_ using a methanizer before detection by a flame ionization detector (FID), CH_4_ was detected by an FID, and N_2_O was detected by an electron capture detector (ECD). The formula for calculating greenhouse gas emission rates is as follows:


$$\begin{array}{c} \begin{array}{c} F=\frac{M}{V}\times H\times \frac{\Delta c}{\Delta t}\times \frac{\text{2}\text{7}\text{3}}{\left(\text{2}\text{7}\text{3}+t\right)} \end{array} \end{array}$$ (1)

where F is the greenhouse gas flux. The fluxes of CO_2_ and CH_4_ are expressed as mg m^-2^ h^-1^, whereas the flux of N_2_O is expressed as μg m^-2^ h^-1^. M is the molar mass of the target gas, with values of 44 g mol^-1^ for CO_2_, 16 g mol^-1^ for CH_4_, and 44 g mol^-1^ for N_2_O. V is the molar volume of gas under standard conditions, taken as 22.4 L mol^-1^. H is the effective chamber height, expressed in m. Δc/Δt represents the rate of change in gas concentration inside the chamber, expressed as μL L^-1^ h^-1^. T is the mean temperature inside the chamber during sampling (℃).

Calculate the cumulative greenhouse gas emissions for each treatment during the growing season using linear interpolation, according to the following formula:


$$\begin{array}{c} \begin{array}{c} CE=\frac{\sum_{i=\text{1}}^{n}\left(F_{i}+F_{i+\text{1}}\right)}{\text{2}}\times \left(t_{i+\text{1}}-t_{i}\right)\times \text{2}\text{4} \end{array} \end{array}$$ (2)

where CE represents the cumulative greenhouse gas emission (kg ha^-1^); F represents the greenhouse gas flux, expressed as mg m^-2^ h^-1^ for CO_2_ and CH_4_ and μg m^-2^ h^-1^ for N_2_O; i is the sampling interval; t represents the number of days between two adjacent sampling dates; n is the number of sampling times, 24 is the correction coefficient.

### Estimation of GWP and GHGI

Global warming potential (GWP, kg CO_2_-eq ha^−1^) is the total exchange of CO_2_, CH_4_ and N_2_O in terms of CO_2_ equivalents. According to the comprehensive greenhouse effect calculation formula for the three types of greenhouse gases over a 100-year time scale as presented in the IPCC report:


$$\begin{array}{c} \begin{array}{c} GWP={CE}_{{co}_{\text{2}}}+{\text{2}\text{8}\times CE}_{{CH}_{\text{4}}}+{\text{2}\text{6}\text{5}\times CE}_{N_{\text{2}}O} \end{array} \end{array}$$ (3)

where CE_CO2_, CE_CH4_ and CE_N2O_ represent the cumulative CO_2_ emissions, cumulative CH_4_ uptake, and cumulative N_2_O emissions during the wheat growing season, respectively. The coefficients 28 and 265 are the 100-year global warming potential factors for CH_4_ and N_2_O relative to CO_2_, respectively. Because CO_2_ exchange measured by static chambers includes soil respiration derived from roots, microorganisms, and residue decomposition, the GWP calculated here represents growing-season soil gas exchange rather than full net ecosystem carbon balance or life-cycle greenhouse gas emissions.

Greenhouse gas intensity (GHGI, kg CO_2_-eq kg^−1^) is the CO_2_ equivalent emissions per grain yield. GHGI was calculated:


$$\begin{array}{c} \begin{array}{c} GHGI=\frac{GWP}{Y} \end{array} \end{array}$$ (4)

where GHGI represents the greenhouse gas emission intensity (kg CO_2_-eq kg^−1^), and Y represents the crop yield (kg ha^-1^).

### Measurement of wheat agronomic traits

At the seedling stage in May 2023, booting stage in May 2023, heading stage in June 2023, and grain-filling stage in June 2023, ten wheat plants were randomly sampled from each plot by cutting them at ground level. Plant height, leaf length, and maximum leaf width were measured using a ruler. Leaf area was calculated based on leaf length, maximum leaf width, and a correction coefficient of 0.83. After the morphological measurements, the fresh weight of each plant was recorded. The samples were then oven-killed at 105℃ for 30 min and subsequently dried at 80℃ to a constant weight. The dry matter weight per plant was determined using an electronic balance. The leaf area index was calculated as follows:


$$\begin{array}{c} \begin{array}{c} LAI=\frac{S}{A} \end{array} \end{array}$$ (5)

where LAI is the leaf area index, expressed as cm^2^·cm^-2^; S is the total leaf area of wheat plants in each plot (cm^2^); and A is the corresponding plot area (cm^2^).

### Wheat yield measurement

At wheat maturity, ten representative plants were randomly sampled from each plot to determine the number of grains per spike. Meanwhile, one 1 m^2^ quadrat was randomly selected from the central area of each plot to avoid edge effects and was used to measure yield components. The number of plants and spikes within each quadrat was recorded, and all spikes in the quadrat were manually harvested. After threshing and air-drying, grain weight was measured and converted to grain yield on a hectare basis. The thousand-grain weight was determined using an electronic balance.

### Statistical analyses

Statistical analyses were performed using IBM SPSS Statistics 19.0. The plot was considered the experimental unit, and data are presented as means of three field replicates. Data normality and homogeneity of variance were checked before analysis. For variables measured once or expressed as cumulative values, including cumulative greenhouse gas emissions, GWP, GHGI, yield components, and grain yield, treatment effects were analyzed using a general linear model appropriate for a randomized block design, with green manure treatment as a fixed factor and block as a random factor. For variables measured repeatedly across growth stages or sampling dates, such as greenhouse gas fluxes, soil temperature, plant height, leaf area index, and dry matter accumulation, treatment effects were analyzed within each sampling date or growth stage. When significant treatment effects were detected, Duncan’s multiple range test was used for mean comparison at P < 0.05. Pearson correlation analysis and linear regression were used to examine relationships among related variables. Figures were prepared using Origin 2021.

## Results

### The effects of different green manure incorporation treatments on soil temperature

As shown in Fig 1, the temporal variation patterns of soil temperature at the 5 cm depth were generally consistent among treatments, indicating that different green manure treatments had relatively limited effects on soil temperature dynamics. With the progression of the spring wheat growing season, soil temperature gradually increased from sowing to harvest. During the wheat growing season, soil temperature ranged from 7.19 to 24.54 ℃ and reached its maximum value on July 12, 2023. Overall, the variation in soil temperature was mainly driven by seasonal climatic conditions and wheat growth processes, resulting in a relatively warmer soil environment during the later growth stages.

**Fig 1:**
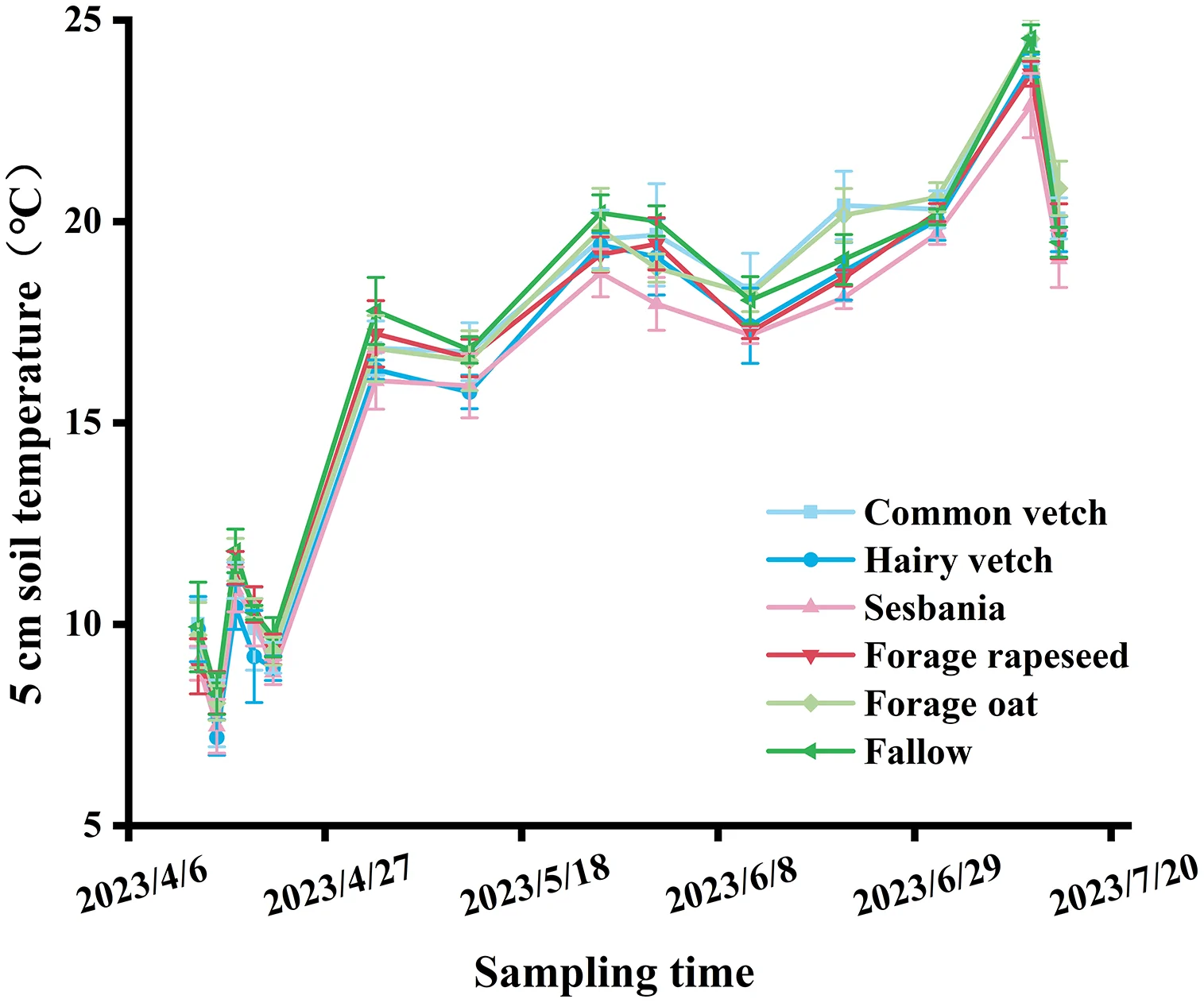
Temporal changes in soil temperature. Soil temperature at 5 cm depth under different green manure incorporation treatments during the spring wheat growing season. Values represent means of three replicates. Soil temperature was measured at each gas sampling event.

### The effects of different green manure incorporation treatments on greenhouse gas emission fluxes

During the growth period of spring wheat, the dynamic changes in soil CO_2_ emission fluxes for each treatment were generally consistent, but different green manure treatments showed significant differences during the key growth stages (Fig 2A). The peak of CO_2_ emissions mainly occurred during the booting stage after fertilization and irrigation. Among them, the CO_2_ emission flux of the sesbania treatment was significantly higher than that of the other treatments (*P* < 0.05), reaching 974.30 mg m^-2^ h^-1^. The CO_2_ emission fluxes for the different treatments during this period were as follows: sesbania > forage rapeseed > fallow > forage oat > hairy vetch > common vetch. After June 2nd, the overall CO_2_ emission fluxes of all treatments showed a downward trend. By the time of wheat filling (June 22nd), the CO_2_ emission fluxes rose again, and the green manure treatments increased by 19.65%−88.28% compared to the fallow treatment. This indicates that green manure incorporation into the soil may continuously affect the soil respiration process during the middle and late growth stages of wheat.

**Fig 2:**
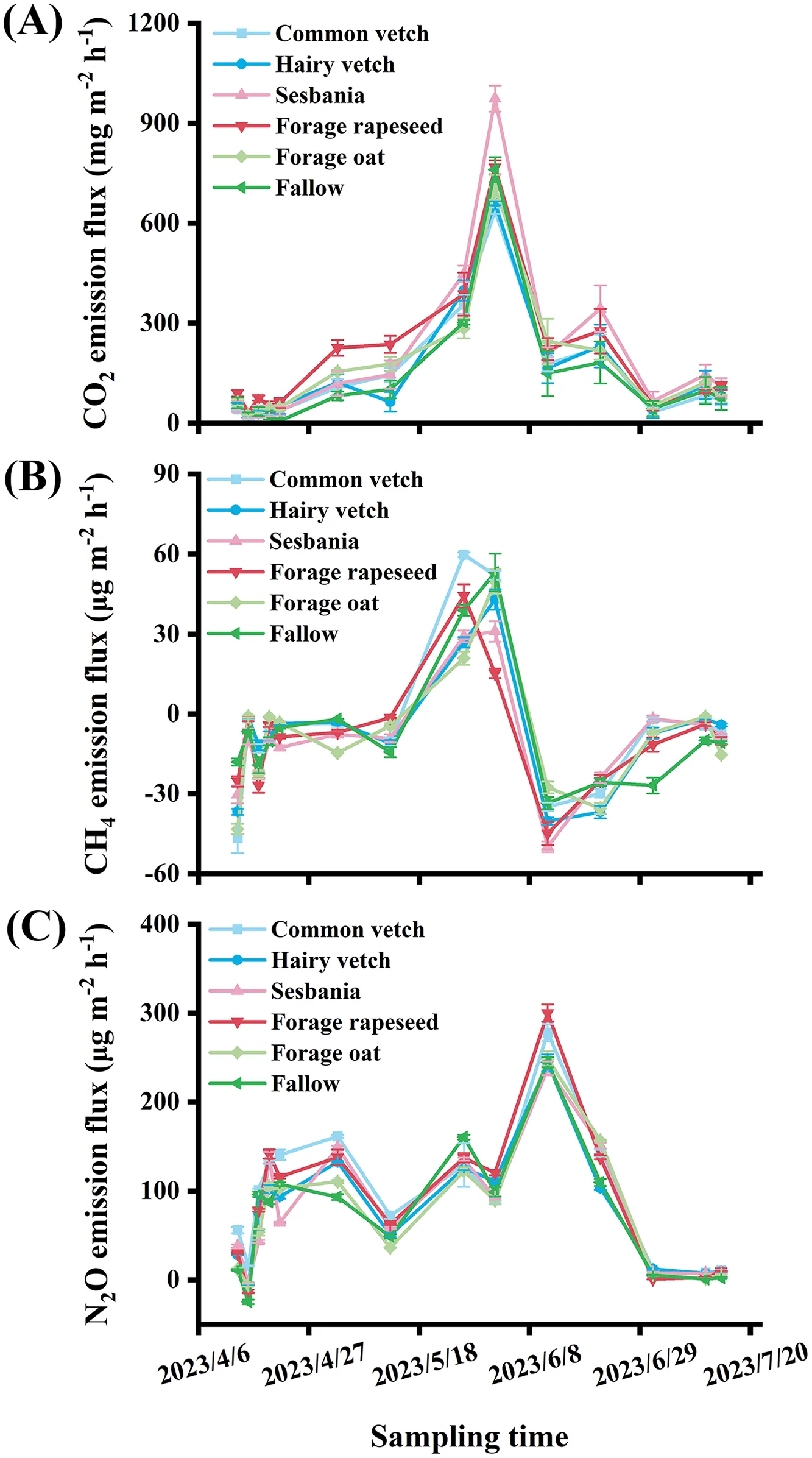
Seasonal patterns of soil greenhouse gas fluxes. Seasonal dynamics of soil greenhouse gas fluxes under different green manure incorporation treatments during the spring wheat growing season. (A) CO_2_ flux; (B) CH_4_ flux; (C) N_2_O flux. Positive CH_4_ values indicate net CH_4_ emission, whereas negative values indicate net CH_4_ uptake.

Soil CH_4_ fluxes showed relatively small fluctuations throughout the wheat growing season, and the temporal variation patterns were generally consistent among treatments (Fig 2B). Overall, the soil acted as a weak CH_4_ source or sink. From the jointing to booting stages (May 27 to June 2), irrigation increased soil moisture, causing CH_4_ fluxes to shift from negative to positive values and reach peak levels. After the heading stage (June 12), CH_4_ uptake gradually approached zero with wheat growth and changes in soil environmental conditions. Overall, cumulative CH_4_ uptake exceeded CH_4_ emissions during the entire wheat growing season, indicating that the wheat field functioned as a weak CH_4_ sink.

The seasonal dynamics of soil N_2_O fluxes were also generally similar among treatments (Fig 2C). During the first 10 d after sowing, N_2_O fluxes initially decreased and then increased, which was likely associated with basal fertilizer application and soil disturbance caused by tillage. Following the first N top-dressing on May 12, N_2_O fluxes were measured on May 13 and May 27, with a marked increase observed on May 27; the forage oat treatment showed the lowest flux (122.68 μg m^-2^ h^-1^). After the second N top-dressing on June 7, N_2_O fluxes increased markedly and reached their highest observed values on June 12, five days after top-dressing. Compared with the fallow treatment, N_2_O fluxes increased by 13.62%, 22.57%, and 1.62% under common vetch, forage rapeseed, and forage oat treatments, respectively, whereas decreases of 0.34% and 2.81% were observed under hairy vetch and sesbania treatments, respectively. Thereafter, N_2_O fluxes gradually declined and stabilized during the late grain-filling stage.

### The effect of different green manure incorporation treatments on cumulative soil greenhouse gas emissions

As shown in Fig 3, different green manure incorporation treatments exerted varying effects on the cumulative emissions of soil CO_2_, CH_4_ and N_2_O. The cumulative CO_2_ emission was highest under the sesbania treatment, reaching 5466.83 kg ha^-1^, whereas the lowest value was observed in the fallow treatment, at 3788.64 kg ha^-1^. Although no significant differences were detected among treatments, all green manure incorporation treatments increased cumulative CO_2_ emissions by 7.29%−44.30% compared with the fallow treatment. This may be attributed to the abundant carbon substrates supplied by green manure incorporation, which stimulated soil CO_2_ emissions (Fig 3A). The cumulative CH_4_ flux indicated that the soil acted as a CH_4_ sink. Except for the common vetch treatment, all green manure incorporation treatments showed higher CH_4_ uptake than the fallow treatment. Among them, the sesbania treatment exhibited the highest CH_4_ uptake, which was significantly increased by 49.65% compared with the fallow treatment. Hairy vetch, forage rapeseed, and forage oat treatments increased CH_4_ uptake by 32.38%, 20.47%, and 21.16%, respectively. In contrast, the common vetch treatment showed a cumulative CH_4_ flux close to zero, with a value of 0.0045 kg ha^−1^, indicating much weaker CH_4_ uptake or a slight net CH_4_ emission (Fig 3B). The cumulative N_2_O emission was highest under the common vetch treatment, reaching 2.51 kg ha^-1^, whereas the lowest value was observed under the forage oat treatment, at 2.06 kg ha^-1^, which was comparable to that under the fallow treatment. Compared with the fallow treatment, common vetch incorporation increased cumulative N_2_O emissions by 21.82%, indicating that different green manure species exerted distinct effects on soil N_2_O emissions (Fig 3C).

**Fig 3:**
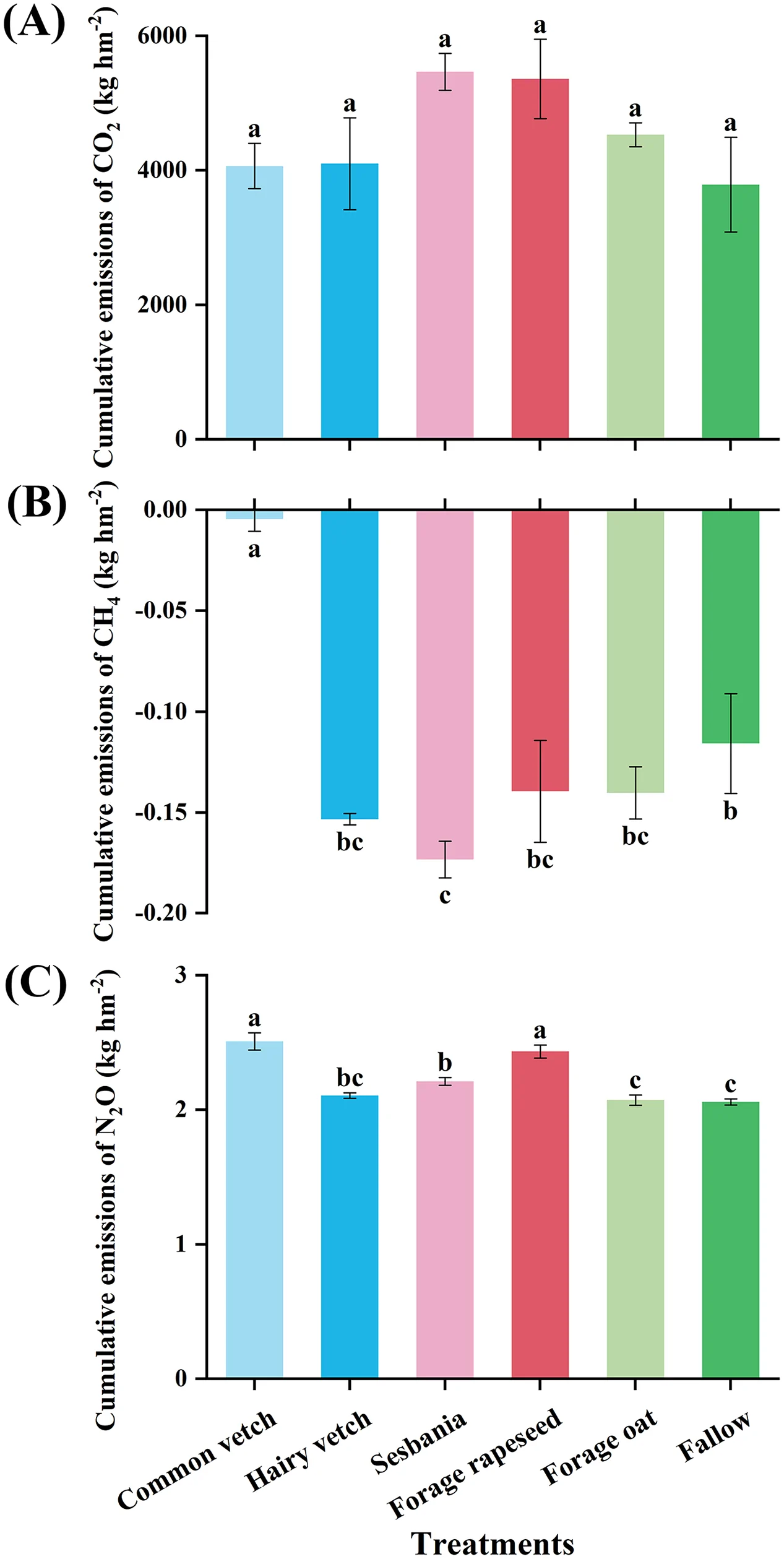
Cumulative soil greenhouse gas emissions during wheat growth. Cumulative soil greenhouse gas emissions under different green manure incorporation treatments during the spring wheat growing season. (A) Cumulative CO_2_ emissions; (B) cumulative CH_4_ fluxes; (C) cumulative N_2_O emissions. Positive CH_4_ values indicate cumulative CH_4_ emission, whereas negative values indicate cumulative CH_4_ uptake.

### Analysis of the correlation between soil temperature and greenhouse gas emission fluxes

As shown in Fig 4, CO_2_ emission fluxes under different green manure incorporation treatments were positively correlated with soil temperature, and this relationship was highly significant (*P* < 0.001). CH_4_ fluxes also showed a weak but significant positive correlation with soil temperature (*P* < 0.05), whereas no significant correlation was observed between N_2_O fluxes and soil temperature. These results indicate that, under the present experimental conditions, soil temperature was closely associated with CO_2_ emission fluxes, but explained only a small proportion of the variation in CH_4_ fluxes. In contrast, N_2_O fluxes were likely regulated more strongly by other factors, such as fertilization, irrigation, soil moisture, and nitrogen substrate availability.

**Fig 4:**
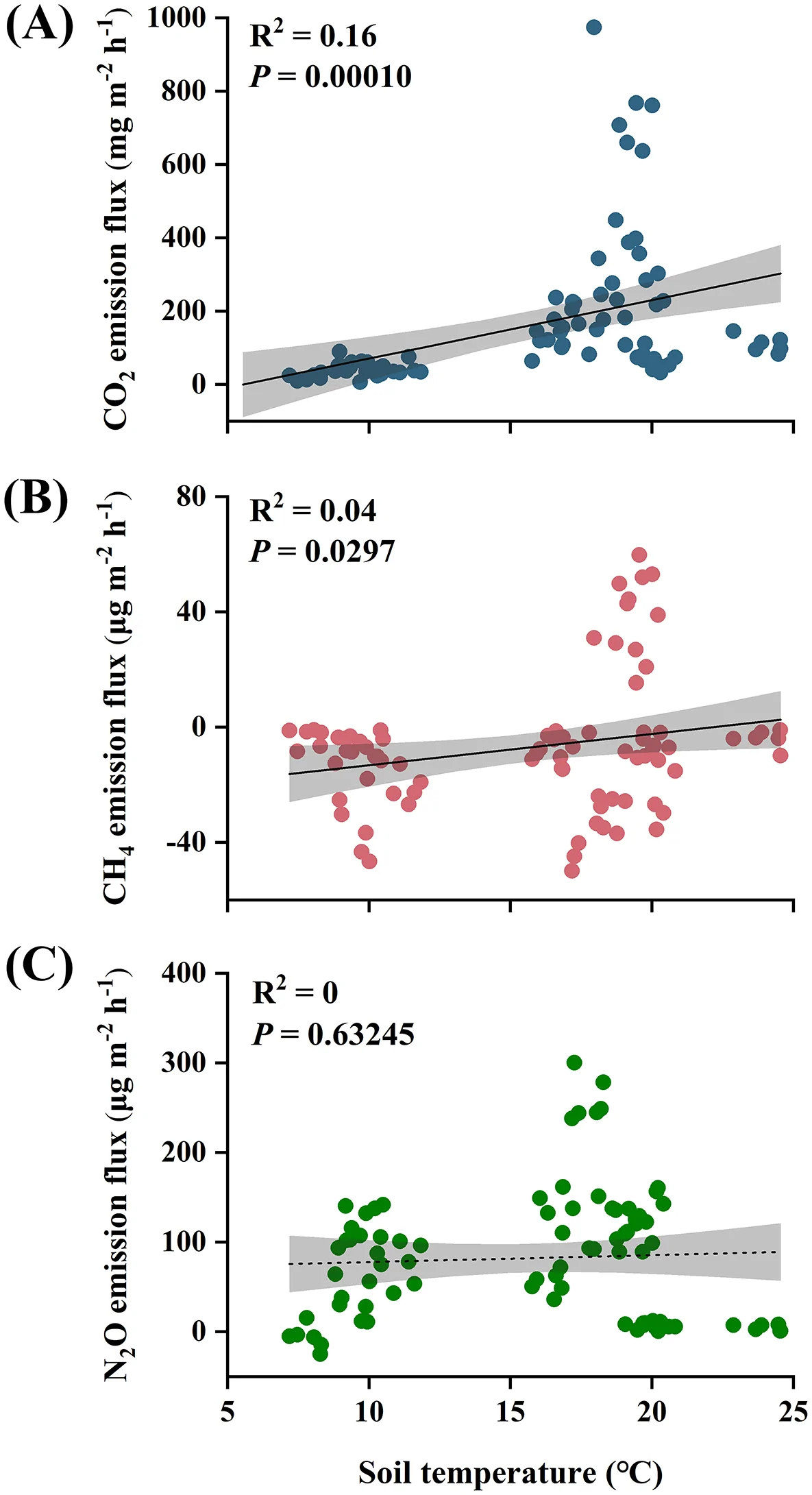
Relationships between soil temperature and greenhouse gas fluxes. (A) relationship between soil temperature and CO_2_ flux; (B) relationship between soil temperature and CH_4_ flux; (C) relationship between soil temperature and N_2_O flux. Solid lines represent fitted linear regression lines, and shaded areas indicate 95% confidence intervals. The coefficient of determination (R^2^) and P value are shown in each panel. Relationships were considered statistically significant at *P* < 0.05.

### Global warming potential (GWP) and greenhouse gas intensity (GHGI)

As shown in Table 1, CO_2_ emissions contributed the most to GWP, whereas CH_4_ fluxes made a negative contribution, thereby partly offsetting the total warming potential. Relative to the fallow treatment, green manure incorporation numerically increased GWP by 7.44%−39.64%, with the largest numerical increases observed under sesbania and forage rapeseed, which increased GWP by 39.64% and 38.56%, respectively. However, treatment differences in GWP were not statistically significant. Across treatments, GHGI followed the order of sesbania > forage rapeseed > forage oat > fallow > common vetch > hairy vetch. Compared with the fallow treatment, common vetch and hairy vetch reduced GHGI, with hairy vetch showing the greatest reduction of 4.10%.

**Table 1:** Effects of green manure incorporation on global warming potential and greenhouse gas emission intensity.

Experimental treatment	GWP (kg CO_2_-eq ha^-1^)	Contribution rate to GWP (%)			GHGI (kg CO_2_-eq kg^-1^)
		CO_2_	CH_4_	N_2_O	
**Common vetch**	4729.94 a	85.94%	0.00%	14.06%	0.97 ab
**Hairy vetch**	4653.52 a	88.09%	−0.09%	12.00%	0.94 b
**Sesbania**	6048.33 a	90.39%	−0.08%	9.69%	1.32 a
**Forage rapeseed**	6001.75 a	89.31%	−0.07%	10.75%	1.21 ab
**Forage oat**	5075.33 a	89.25%	−0.08%	10.83%	1.03 ab
**Fallow**	4331.42 a	87.47%	−0.07%	12.61%	0.98 ab

Note: Different lowercase letters indicate significant differences among different treatments (*P* < 0.05).

### The effects of different green manure crops on the agronomic traits of wheat

#### The effect of different green manure crops on wheat plant height.

As shown in Fig 5, wheat plant height increased progressively with crop development. The increase was rapid before the booting stage, but slowed after heading, and plant height tended to stabilize during the grain-filling stage. At the seedling stage, the forage rapeseed and forage oat treatments showed the greatest plant height, with values significantly higher than those of the other treatments (*P* < 0.05). At the booting stage, plant height under the hairy vetch treatment was 4.44% higher than that under the fallow treatment, although no significant differences were observed among treatments at this stage. At the heading stage, plant height was higher under all green manure treatments than under the fallow treatment, except for hairy vetch. During the grain-filling stage, wheat growth slowed and plant height became relatively stable. Compared with the fallow treatment, green manure incorporation increased plant height by 3.68%−11.32%, and the final plant height was reached after grain filling and maturity.

**Fig 5:**
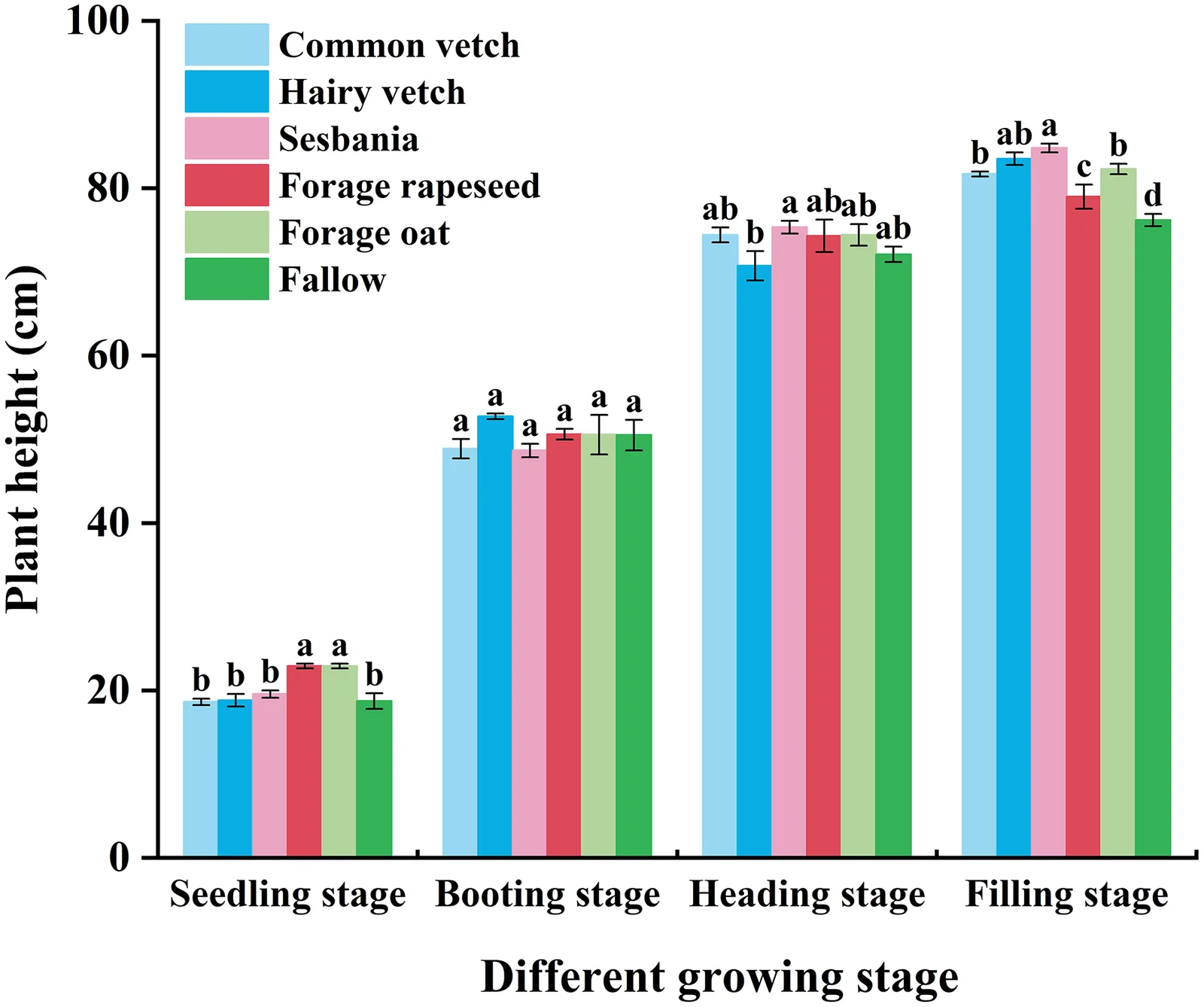
Wheat plant height across growth stages. Changes in wheat plant height under different green manure incorporation treatments across growth stages. Values represent means ± standard errors of three replicates. Different lowercase letters indicate significant differences among treatments within the same growth stage at *P* < 0.05.

#### The effect of different green manure crops on leaf area index.

As shown in Fig 6, wheat leaf area index (LAI) varied markedly among treatments during the growth period. Overall, LAI increased at the early growth stages and then declined, reaching its maximum around the booting stage. At the seedling stage, LAI under the forage oat and forage rapeseed treatments was significantly higher than that under the fallow treatment by 29.39% and 19.28%, respectively (*P* < 0.05). At the booting stage, LAI peaked in all treatments and followed the order of forage rapeseed > forage oat > hairy vetch > fallow > common vetch > sesbania. The forage rapeseed, forage oat, and hairy vetch treatments significantly increased LAI compared with the fallow treatment. After booting, LAI began to decline in most treatments, except for common vetch and sesbania. After heading, LAI decreased across all treatments. At the grain-filling stage, green manure incorporation increased LAI by 4.94%−52.60% relative to the fallow treatment. In particular, forage oat, common vetch, and forage rapeseed had significantly higher LAI than hairy vetch, sesbania, and fallow (*P* < 0.05).

**Fig 6:**
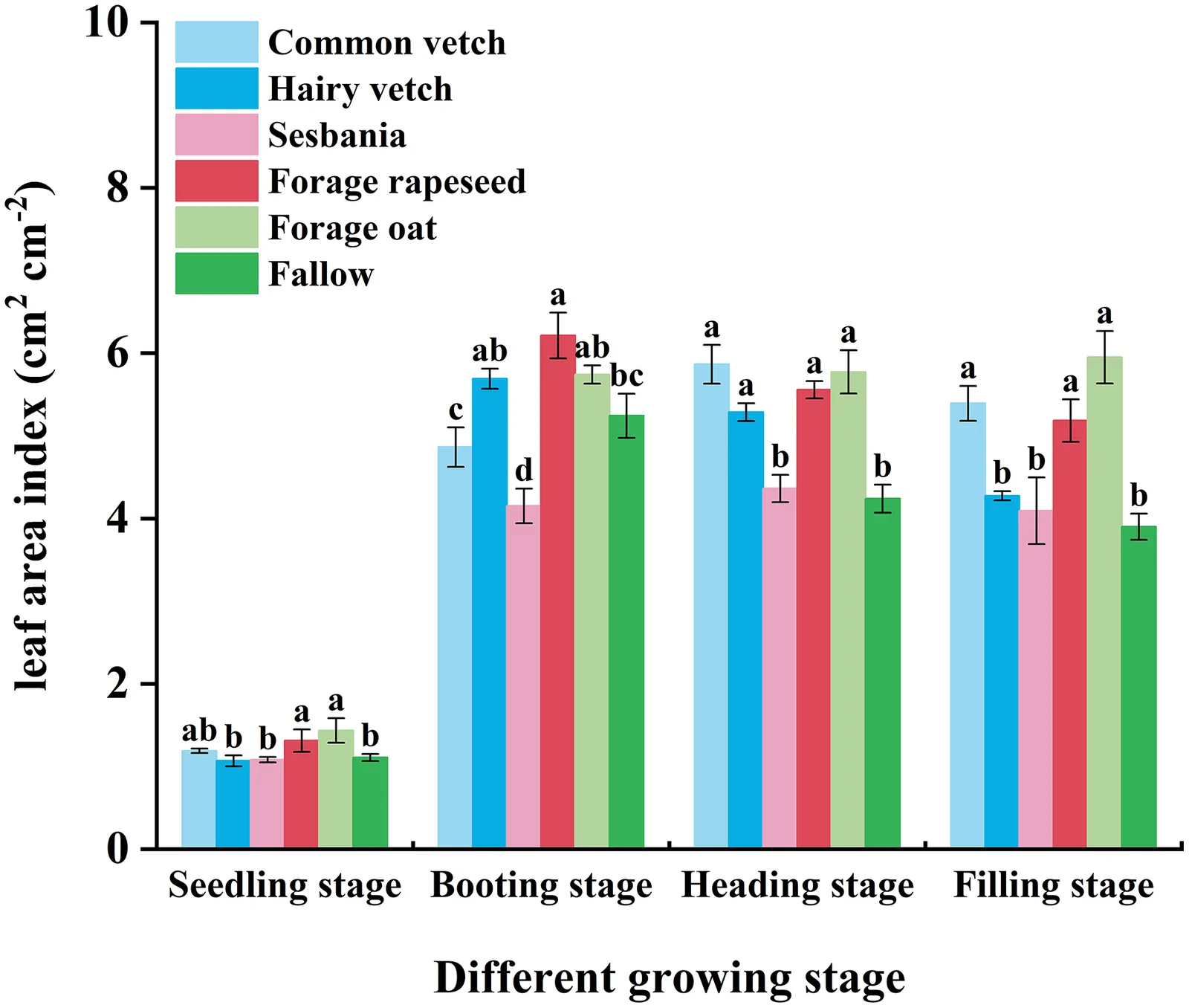
Wheat leaf area index across growth stages. Changes in wheat leaf area index under different green manure incorporation treatments across growth stages. Values represent means ± standard errors of three replicates. Different lowercase letters indicate significant differences among treatments within the same growth stage at *P* < 0.05.

#### The effect of different green manure crops on dry matter accumulation per plant of wheat.

As shown in Fig 7, aboveground dry matter accumulation in wheat showed a broadly similar trend across treatments during the growth period. It increased progressively with crop development and reached the maximum at the grain-filling stage. At the seedling stage, wheat growth was slow, and dry matter accumulation remained low. From the booting to heading stages, wheat growth accelerated with the seasonal increase in temperature, resulting in a continuous increase in aboveground dry matter accumulation. At the heading stage, the sesbania treatment showed the most rapid increase, with aboveground dry matter accumulation significantly higher than that of the fallow treatment by 24.03%. At the grain-filling stage, all green manure treatments significantly increased aboveground dry matter accumulation compared with the fallow treatment, with increases ranging from 19.10% to 50.11% (*P* < 0.05). The fallow treatment showed the lowest dry matter accumulation at this stage.

**Fig 7:**
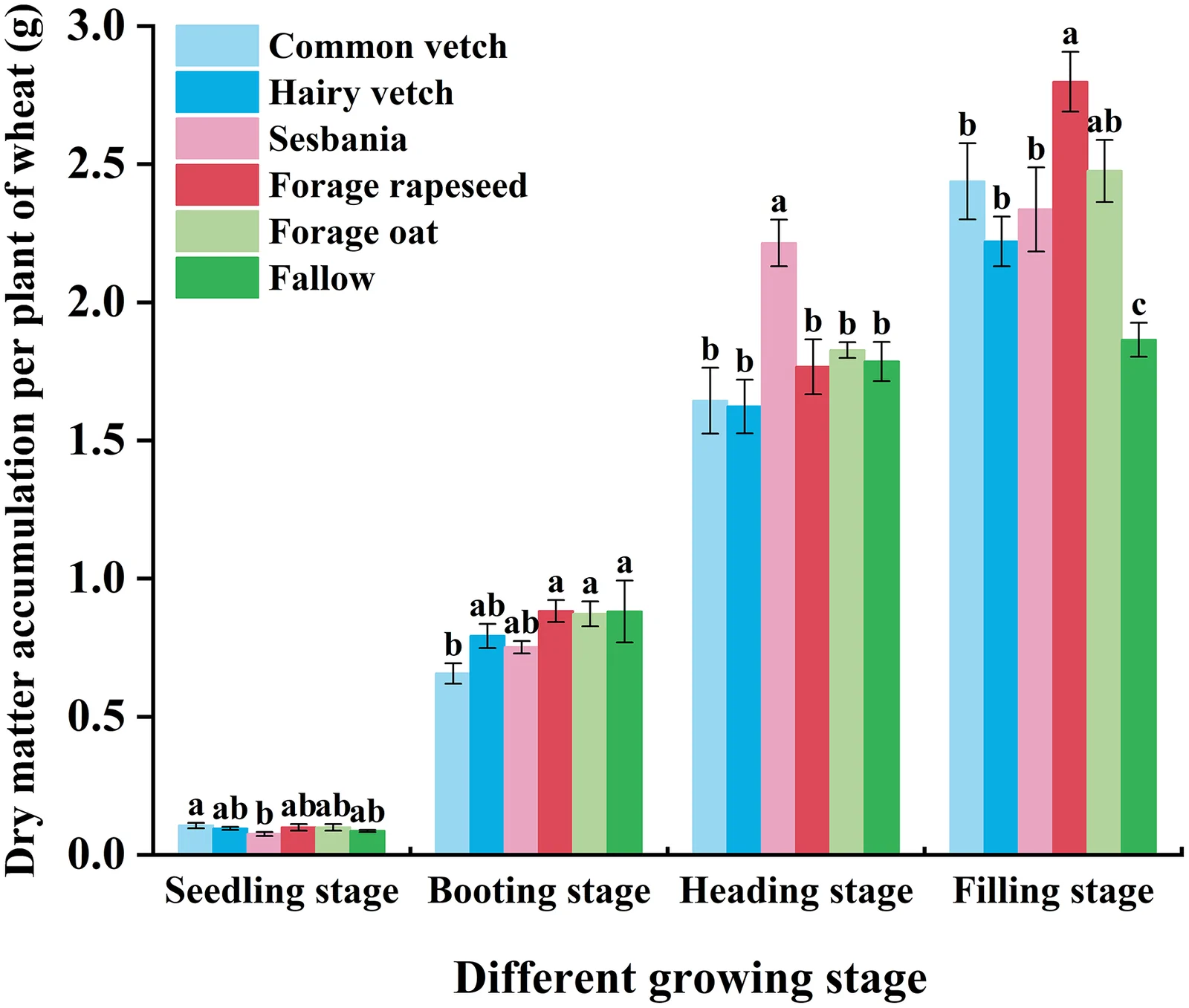
Wheat dry matter accumulation across growth stages. Changes in aboveground dry matter accumulation of wheat under different green manure incorporation treatments across growth stages. Values represent means ± standard errors of three replicates. Different lowercase letters indicate significant differences among treatments within the same growth stage at *P* < 0.05.

### The effects of different green manure crops on wheat yield

As shown in Table 2, green manure incorporation had highly significant effects on the effective spike number, grain number per spike, 1000-grain weight, and grain yield of wheat. The effective spike number followed the order of hairy vetch > forage oat > forage rapeseed > common vetch > fallow > sesbania. Compared with the fallow treatment, hairy vetch, forage oat, forage rapeseed, and common vetch increased the effective spike number by 22.18%, 15.27%, 14.40%, and 8.87%, respectively. The grain number per spike under the forage oat treatment was 25.36% higher than that under the fallow treatment. Compared with the fallow treatment, common vetch, hairy vetch, forage rapeseed, and forage oat increased 1000-grain weight by 23.08%, 25.84%, 34.19%, and 23.92%, respectively. After green manure incorporation following the 2022 wheat season, wheat grain yield increased under all green manure treatments compared with the fallow treatment. The highest grain yield was observed under the hairy vetch treatment, which was 11.95% higher than that under the fallow treatment. Overall, green manure incorporation increased wheat yield by 2.96%−11.95%. The harvest index followed the order of sesbania > common vetch > hairy vetch > fallow > forage rapeseed > forage oat. These results indicate that the yield increase induced by green manure incorporation was mainly associated with improvements in effective spike number and 1000-grain weight. Green manure incorporation promoted high yield by maintaining a relatively high spike number per unit area while increasing grain number per spike and grain weight.

**Table 2:** Effect of green manure incorporation on wheat yield.

Treatment	Effective spike number (×10^4^ spikes ha^-1^)	Kernel number per spike	1000-kernel weight (g)	Yield (kg ha^-1^)
**Common vetch**	623.75 b	39.70 c	40.96 a	4879.50 a
**Hairy vetch**	700.00 a	39.40 c	41.88 a	4985.31 a
**Sesbania**	568.75 c	41.90 b	33.18 b	4584.97 b
**Forage rapeseed**	655.42 ab	39.40 c	44.66 a	4953.97 a
**Forage oat**	660.42 ab	44.00 a	41.24 a	4947.36 a
**Fallow**	572.92 c	35.10 d	33.28 b	4453.29 b

Note: Different lowercase letters within the same column indicate a statistically significant difference at the 5% level (*P* < 0.05).

### Correlation between wheat yield and various indicators

As shown in Fig 8, Pearson correlation analysis showed that wheat grain yield was highly significantly and positively correlated with effective spike number (R = 0.92, *P* < 0.01) and 1000-kernel weight (R = 0.95, *P* < 0.01). In addition, 1000-kernel weight was significantly correlated with effective spike number (R = 0.87, *P* < 0.05). Cumulative CH_4_ flux was negatively correlated with cumulative CO_2_ emissions, plant height, effective spike number, and kernel number per spike, although these correlations were not significant.

**Fig 8:**
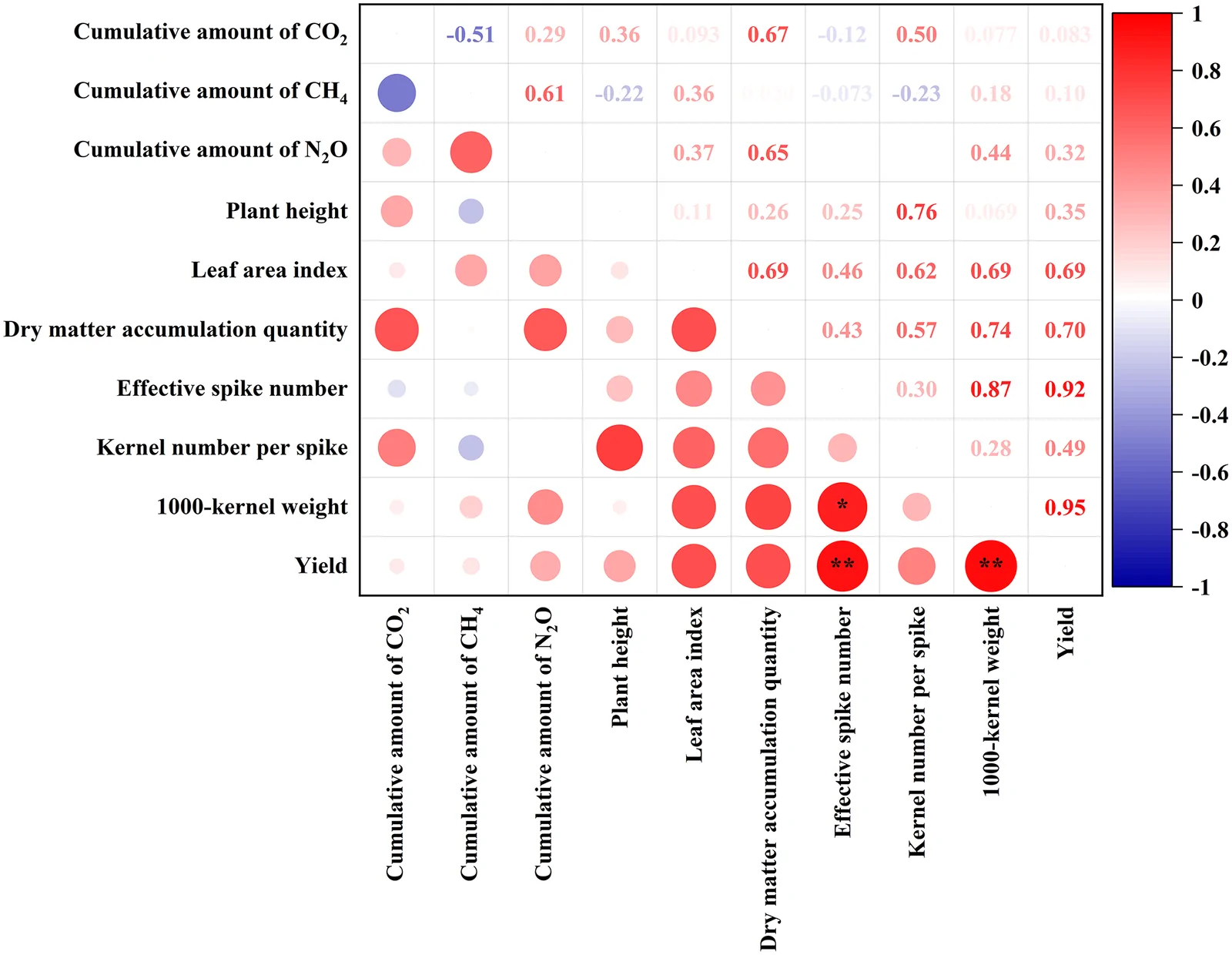
Correlations among yield and greenhouse gas indicators. The color scale represents the strength and direction of Pearson correlation coefficients. Asterisks indicate statistical significance: * indicates *P* < 0.05, ** indicates *P* < 0.01. CO_2_, CH_4_, and N_2_O represent cumulative greenhouse gas fluxes or emissions during the spring wheat growing season.

## Discussion

### The impact of different green manure incorporation treatments on soil greenhouse gas emissions and the overall greenhouse effect

This study indicates that the impact of green manure incorporation on greenhouse gas emissions in wheat fields shows significant differences by gas type and green manure species. This suggests that its environmental effect cannot be simply summarized as either increased or decreased emissions [6]. After incorporation, green manure can alter soil carbon and nitrogen substrate availability, microbial activity, and soil hydrothermal conditions through the input of organic carbon and nitrogen, thereby affecting CO_2_, N_2_O, and CH_4_ emission processes [18]. Similar studies have also shown that exogenous organic carbon inputs can regulate soil carbon and nitrogen cycling and greenhouse gas emissions in farmland, but their effects are strongly influenced by amendment type, soil hydrothermal conditions, and crop growth [19]. Previous studies have also demonstrated greenhouse gas mitigation benefits of some green manure species evaluated in the present study under appropriate management conditions. In an arid irrigated maize system, intercropping with common vetch reduced both cumulative and yield-scaled N_2_O emissions, particularly when combined with reduced chemical N input [20]. Partial substitution of urea N with oilseed rape green manure also reduced GWP and GHGI without compromising crop yield [21]. In addition, oat cover cropping reduced N_2_O emissions relative to fallow under an organic cotton system [22]. Together, these studies suggest that species- and management-dependent differences in carbon and nitrogen inputs, as well as soil environmental conditions, may underlie the contrasting greenhouse gas responses to green manure. Therefore, the greenhouse gas responses to green manure incorporation should be interpreted from three linked processes: carbon turnover, nitrogen transformation, and soil aeration.

Studies have shown that the most direct effect of green manure incorporation into the soil is to increase the input of organic carbon in the soil, thereby enhancing the soil respiration process. The main sources of CO_2_ in farmland soil are plant root respiration, soil microbial respiration, soil animal respiration, and the chemical oxidation process of carbon-containing substances [23]. In this study, cumulative CO_2_ emissions under green manure treatments increased by 7.29%−44.30% compared with the fallow treatment, with the highest value observed under sesbania. This indicates that green manure residues supplied abundant carbon and nitrogen substrates for microbial activity, thereby stimulating soil respiration [24]. The increase in CO_2_ flux after fertilization and irrigation, together with the highly significant positive correlation between CO_2_ flux and soil temperature, suggests that moisture, nutrient availability, and temperature jointly promoted green manure decomposition and microbial metabolism [25]. Wang et al. also reported that CO_2_ and N_2_O fluxes often peaked after fertilization in mulched farmland, mainly because fertilizer inputs supplied substrates and hydrothermal conditions promoted soil carbon and nitrogen transformations [19]. Muhammad et al. reported that cover crop residues generally increased CO_2_ emissions, whereas leguminous cover crops were more likely to increase N_2_O emissions; moreover, residue incorporation into soil enhanced both CO_2_ and N_2_O emissions compared with surface placement or residue removal [26]. However, it should be noted that an increase in short-term CO_2_ emissions does not necessarily mean a decline in the long-term carbon sequestration potential. This is because green manuring and returning of crop residues also increase the input of litter carbon and root carbon. Its long-term carbon effect depends on the balance among carbon input, CO_2_ release, and soil organic carbon accumulation [27].

While the carbon cycle is being enhanced, the nitrogen release from green manure residues further affects the process of N_2_O production. N_2_O is mainly produced by soil microorganisms through nitrification and denitrification processes, including autotrophic nitrification, nitrifier denitrification, heterotrophic denitrification, and co-denitrification [28]. Growing green manure or cover crops during the fallow period can absorb residual soil nitrogen, thereby reducing nitrogen loss and potential N_2_O emission risk [29]. However, after incorporation, residues with a low C/N ratio can mineralize rapidly, increase soil available nitrogen, and provide substrates for nitrification and denitrification, thus promoting N_2_O emissions [30,31]. In this study, cumulative N_2_O emissions were highest under common vetch and lowest under forage oat, which may be related to the faster nitrogen release from leguminous green manure and the stronger short-term nitrogen immobilization associated with gramineous green manure, respectively [30]. Liu et al. also reported in rainfed wheat that soil available nitrogen is an important factor affecting cumulative N_2_O emissions, and that N_2_O emission peaks tend to occur after fertilization and rainfall events [32]. Consistent with this pattern, the highest observed N_2_O flux in our study occurred five days after the second N fertilization. This response may be associated with increased N availability following fertilization, which may have promoted soil N transformation and subsequent N_2_O production [31,32]. Notably, hairy vetch, although also a leguminous green manure, did not show the highest N_2_O emissions. This indicates that the emission risk of leguminous green manure depends not only on residue nitrogen content, but also on the synchrony between green manure nitrogen release and wheat nitrogen uptake. Previous studies have shown that mixed cropping of hairy vetch and common vetch can reduce N_2_O emissions from wheat fields, which may be related to biological nitrogen fixation, regulation by root exudates, and crop-microbe competition for nitrogen [11].

Unlike CO_2_ and N_2_O, which are mainly regulated by carbon and nitrogen substrate supplies, CH_4_ flux is more dependent on soil aeration and the methane oxidation process. CH_4_ flux is controlled by the relative strength of methanogenesis and methane oxidation. When methanogenesis exceeds methane oxidation, soil acts as a CH_4_ source; conversely, when methane oxidation dominates, soil acts as a CH_4_ sink [33]. In this study, the wheat field generally acted as a CH_4_ sink, and all green manure treatments except common vetch increased CH_4_ uptake, with the highest uptake observed under sesbania. This suggests that methane oxidation predominated in the upland wheat field. Green manure incorporation may enhance CH_4_ uptake by improving soil structure and stimulating methanotrophic activity [34]. However, increased soil moisture after irrigation or rainfall can restrict gas diffusion and create localized anaerobic microsites, resulting in weakened CH_4_ uptake or even short-term CH_4_ emissions [35]. Previous studies have also indicated that CH_4_ uptake is closely related to soil aeration, moisture status, and methane oxidation processes, and that NH_4_^+^ after fertilization may weaken CH_4_ oxidation by affecting methane monooxygenase activity [19]. In this study, CH_4_ flux showed a weak but significant relationship with soil temperature. However, the low R^2^ value indicated that soil temperature explained only a small proportion of the variation in CH_4_ fluxes. Therefore, CH_4_ uptake was still more likely regulated by soil moisture, aeration conditions, and methanotrophic activity rather than by temperature alone [36].

In summary, the contrasting responses of CO_2_, N_2_O, and CH_4_ jointly determined the changes in GWP and GHGI among green manure treatments. In this study, CO_2_ was the dominant contributor to GWP, whereas CH_4_ uptake made a negative but limited contribution because of its relatively small flux magnitude. Consequently, although most green manure treatments enhanced CH_4_ uptake, GWP still numerically increased by 7.44%−39.64% compared with the fallow treatment, with the greatest numerical increases observed under sesbania and forage rapeseed. This suggests that, in the short term, green manure residue decomposition may have intensified soil carbon and nitrogen turnover, thereby increasing area-scaled warming effects through enhanced CO_2_ release and, in some treatments, increased N_2_O emissions [24]. However, a higher GWP does not necessarily indicate poorer environmental performance, because GHGI also depends on the extent to which yield gains compensate for increased emissions [37]. Previous studies have suggested that GHGI is a useful yield-scaled indicator for evaluating the trade-off between crop productivity and greenhouse gas emissions in wheat production systems [38]. Although sesbania showed the highest CH_4_ uptake, its high cumulative CO_2_ emissions and GWP were not sufficiently offset by yield improvement, resulting in the highest GHGI. In contrast, hairy vetch did not show the lowest GWP, but it produced the highest wheat yield and the lowest GHGI, suggesting a more efficient conversion of green manure-derived resources into grain yield. Similar trade-offs among yield, environmental costs, and carbon or nitrogen footprints have also been reported in semi-arid wheat systems [39]. Moreover, practices that enhance soil carbon accumulation may reduce yield-scaled carbon footprints, even when field greenhouse gas emissions are not the lowest [40]. Therefore, under the present experimental conditions, hairy vetch showed the strongest capacity to coordinate wheat yield improvement with lower yield-scaled greenhouse gas emissions. Meanwhile, common vetch also reduced GHGI relative to the fallow treatment, and forage oat showed the lowest cumulative N_2_O emissions with a marked yield increase. These results indicate that different green manure species contributed to low-carbon and high-yield production through different pathways.

### Effects of different green manure species on wheat growth, development, and yield formation

The positive effect of green manure incorporation on wheat yield formation was not simply attributable to nutrient supplementation, but was achieved through the combined improvement of soil functioning, nitrogen supply, wheat vegetative growth and leaf area development [29,41]. In this study, green manure incorporation generally increased wheat plant height, leaf area index, aboveground dry matter accumulation, and grain yield, indicating that green manure replanting after wheat harvest improved vegetative growth and leaf area development in the subsequent wheat crop. Specifically, plant height under green manure treatments increased by 3.68%−11.32% compared with the fallow treatment at the grain-filling stage, and leaf area index reached its peak around the booting stage, with forage rapeseed, forage oat, and hairy vetch showing significantly higher values than the fallow treatment at this stage. These results indicate that green manure incorporation enhanced leaf area development and assimilate production capacity. Wu et al. suggested that leaf area, dry matter accumulation, and grain sink capacity jointly determine final yield [42]. Therefore, the improvement in leaf area under green manure treatments provided a physiological basis for subsequent yield formation.

Whether the advantage in leaf area can be translated into yield further depends on dry matter accumulation during the middle and late growth stages and its allocation to grains. In this study, aboveground dry matter accumulation increased continuously with wheat development and reached its maximum at the grain-filling stage. At this stage, all green manure treatments significantly increased dry matter accumulation by 19.10%−50.11% compared with the fallow treatment, indicating that green manure incorporation enhanced assimilate production during the middle and late growth stages. Duan et al. reported that pre-anthesis dry matter and nitrogen accumulation are closely associated with grain number formation in wheat and represent an important basis for yield formation [43]. In this study, wheat grain yield under green manure treatments increased by 2.96%−11.95% compared with the fallow treatment, with the greatest increases observed under hairy vetch, forage rapeseed, and forage oat, reaching 11.95%, 11.24%, and 11.09%, respectively. Previous studies have also shown that wheat yield improvement is closely associated with assimilate accumulation, translocation efficiency, and optimization of yield components such as grain number per spike, 1000-kernel weight, and harvest index [44]. These results indicate that the increase in dry matter accumulation provided the material basis for yield improvement, whereas the final yield differences among treatments were further determined by the responses of specific yield components.

Furthermore, the differences in effective spike number, kernel number per spike, and 1000-kernel weight indicate that different green manure species regulated wheat yield formation through different pathways. The yield differences among green manure treatments further indicate that the synchrony between nutrient release from green manure residues and wheat nutrient demand is a key factor determining yield response [11]. In this study, hairy vetch produced the highest effective spike number and grain yield, suggesting that it may have better coordinated nutrient release and grain filling. Wei et al. showed that legume green manure can enhance subsequent wheat productivity by increasing nitrogen supply and wheat nitrogen uptake, providing a mechanistic explanation for the higher yield observed under hairy vetch [9]. Forage oat produced the highest kernel number per spike and also markedly increased grain yield, indicating that gramineous green manure can promote yield formation by increasing kernel number per spike. Forage rapeseed produced the highest 1000-kernel weight, suggesting a stronger positive effect on grain filling. Therefore, hairy vetch, forage oat, and forage rapeseed promoted yield formation through advantages in effective spike number, kernel number per spike, and 1000-kernel weight, respectively, while green manure species selection should integrate yield components, dry matter accumulation, GHGI, and N_2_O emissions [37,38]. Overall, green manure incorporation promoted wheat yield formation by improving vegetative growth, enhancing dry matter accumulation, and optimizing yield components. Among the selected green manure varieties, hairy vetch showed the best performance in coordinating yield improvement with lower yield-scaled greenhouse gas emissions, while forage oat and common vetch also showed potential advantages in yield enhancement and lower GHGI, respectively. Therefore, green manure species selection should integrate yield components, dry matter accumulation, GHGI, and N_2_O emissions rather than relying only on grain yield.

### Integrated assessment and implications for low-carbon and high-yield green manure management

The preceding sections indicate that green manure incorporation generated a clear production-environment trade-off in the spring wheat system. Green manure treatments improved wheat yield formation through enhanced vegetative growth, dry matter accumulation, and yield-component performance, but also tended to increase soil-exchange-based GWP through intensified soil carbon and nitrogen turnover. Therefore, green manure species should be evaluated not by a single agronomic trait or individual greenhouse gas response, but by the extent to which yield gains compensate for increased warming effects. This evaluation logic is consistent with the principle of producing more grain with lower environmental costs and with the need to balance high yield and greenhouse gas mitigation in intensive wheat systems [3].

Under this integrated framework, different green manure species showed distinct functional advantages. Hairy vetch had the strongest overall performance, as it combined the highest grain yield with the lowest GHGI, indicating a high capacity to coordinate productivity with lower yield-scaled greenhouse gas emissions. Common vetch reduced GHGI relative to the fallow treatment despite its higher N_2_O emissions, suggesting that its yield compensation partially offset the environmental cost of increased N_2_O release. Forage oat combined a marked yield increase with the lowest cumulative N_2_O emission, indicating its suitability for systems aiming to improve yield while constraining N_2_O risk. In contrast, sesbania and forage rapeseed showed weaker low-carbon advantages because their higher GWP or GHGI limited the environmental benefits associated with yield improvement. These results suggest that green manure application should shift from selecting a universally optimal species to matching species with specific production and mitigation targets.

From an application perspective, green manure management in spring wheat systems should move toward the co-optimization of productivity, GWP control, and GHGI reduction. Hairy vetch is more suitable for high-yield and low-GHGI objectives, common vetch may be considered where reducing yield-scaled emission intensity is prioritized, and forage oat may be useful where N_2_O mitigation is a key concern. Because irrigation and nitrogen management strongly affect greenhouse gas emissions while maintaining crop productivity, green manure application should be integrated with water and nitrogen optimization rather than implemented as an isolated practice [45]. Long-term field studies are still needed to evaluate whether these short-term advantages persist in terms of soil organic carbon accumulation, nitrogen-use efficiency, yield stability, and economic returns.

## Conclusion

This study showed that the spring wheat system acted as a source of CO_2_ and N_2_O emissions and a weak sink for CH_4_. Compared with the fallow treatment, green manure incorporation increased cumulative CO_2_ emissions by 7.29%−44.30% and enhanced CH_4_ uptake in most treatments. Green manure incorporation numerically increased growing-season soil-exchange-based GWP by 7.44%−39.64%, although treatment differences in GWP were not statistically significant. CO_2_ contributed more than 85% of total soil-exchange-based GWP. Meanwhile, green manure incorporation promoted wheat growth and yield formation. At the grain-filling stage, plant height, leaf area index, and aboveground dry matter accumulation increased by 3.68%−11.32%, 4.94%−52.60%, and 19.10%−50.11%, respectively, compared with the fallow treatment. Wheat grain yield increased by 2.96%−11.95%, with the greatest increases observed under hairy vetch, forage rapeseed, and forage oat. Overall, hairy vetch showed the best coordination between yield improvement and lower GHGI, common vetch numerically reduced GHGI relative to fallow despite higher N_2_O emissions, and forage oat combined relatively high yield with the lowest cumulative N_2_O emissions. Therefore, under the present experimental conditions, hairy vetch, common vetch, and forage oat can be considered promising green manure species for incorporation after spring wheat harvest in the Yellow River Basin of Inner Mongolia. However, their long-term effects on soil carbon accumulation, nitrogen-use efficiency, yield stability, and full life-cycle greenhouse gas balance require further evaluation.

## Supporting information

S1 Dataset Data used for statistical analyses and figure generation. (XLSX)

S1 Table Nutrient contents and reported soil pH ranges of different green manure species. (DOCX)

S2 Table Green manure biomass incorporated and nutrient inputs to the soil in different treatments. (DOCX)
