# Supplementary material for: Species-specific effects of post-wheat green manure incorporation on spring wheat yield and soil greenhouse gas emissions in an arid irrigated system
Source: PLoS One. 2026 Oct 14;21(10):e0360932. doi: 10.1371/journal.pone.0360932 (PMC13667824; doi:10.1371/journal.pone.0360932)
Supplement: Supplementary file 1 [file pone.0360932.s002.docx]

**S1 Table. Nutrient contents and reported soil pH conditions of different green manure species.**

| **Green manure** | **N (g kg^-1^)** | **P (g kg^-1^)** | **K (g kg^-1^)** | **C/N** | **Soil pH conditions for growth** |
| --- | --- | --- | --- | --- | --- |
| **Common vetch** | 10.41 ± 0.63 a | 3.23 ± 0.07 bc | 6.30 ± 0.09 d | 33.36 ± 1.38 c | 4.5-9.0 ^[1]^ |
| **Hairy vetch** | 10.52 ± 0.16 a | 3.01 ± 0.25 c | 7.32 ± 0.05 c | 34.86 ± 0.77 c | 6.1-9.7 ^[2]^ |
| **Sesbania** | 4.78 ± 0.07 c | 4.69 ± 0.25 a | 9.87 ± 0.10 a | 67.28 ± 1.00 b | 7.5-8.7 ^[3]^ |
| **Forage rapeseed** | 9.43 ± 0.3 b | 3.67 ± 0.20 b | 9.34 ± 0.18 b | 31.97 ± 0.74 c | 5.5-8.5 ^[4]^ |
| **Forage oat** | 2.80 ± 0.03 d | 2.95 ± 0.38 c | 5.42 ± 0.15 e | 141.88 ± 5.71 a | up to 9.0 ^[5]^ |

Note: Different lowercase letters indicate significant differences among different green manure species (*P* < 0.05). References [1-5] correspond to the reported soil pH conditions for growth of the respective green manure species.

[1] Huang YF, Gao XL, Nan ZB, Zhang ZX. Potential value of the common vetch (*Vicia sativa* L.) as an animal feedstuff: a review. J Anim Physiol Anim Nutr. 2017;101(5):807-823. doi: 10.1111/jpn.12617.

[2] Renzi JP, Chantre GR, Smýkal P, Presotto AD, Zubiaga L, Garayalde AF, et al. Diversity of naturalized hairy vetch (*Vicia villosa* Roth) populations in Central Argentina as a source of potential adaptive traits for breeding. Front Plant Sci. 2020;11:189. doi: 10.3389/fpls.2020.00189.

[3] Zheng H, Wang X, Chen L, Wang Z, Xia Y, Zhang Y, et al. Enhanced growth of halophyte plants in biochar-amended coastal soil: roles of nutrient availability and rhizosphere microbial modulation. Plant Cell Environ. 2018;41(3):517-532. doi: 10.1111/pce.12944.

[4] Jones C, Olson-Rutz K. Soil nutrient management for canola. Bozeman (MT): Montana State University Extension; 2024. EB0224.

[5] Bai J, Yan W, Wang Y, Yin Q, Liu J, Wight C, Ma B. Screening oat genotypes for tolerance to salinity and alkalinity. Front Plant Sci. 2018;9:1302. doi:10.3389/fpls.2018.01302.
