# Supplementary material for: Species-specific effects of post-wheat green manure incorporation on spring wheat yield and soil greenhouse gas emissions in an arid irrigated system
Source: PLoS One. 2026 Oct 14;21(10):e0360932. doi: 10.1371/journal.pone.0360932 (PMC13667824; doi:10.1371/journal.pone.0360932)
Supplement: Supplementary file 2 [file pone.0360932.s003.docx]

**S2 Table. Green manure biomass incorporated and nutrient inputs to the soil in different treatments.**

| **Green manure** | **Biomass incorporated (kg ha^-1^)** | **N input (kg ha^-1^)** | **P input (kg ha^-1^)** | **K input (kg ha^-1^)** |
| --- | --- | --- | --- | --- |
| **Common vetch** | 5076.27 ± 31.05 b | 52.82 ± 3.07 a | 16.40 ± 0.36 c | 31.96 ± 0.46 c |
| **Hairy vetch** | 5343.68 ± 37.89 b | 56.20 ± 0.48 a | 16.10 ± 1.39 c | 39.12 ± 0.51 b |
| **Sesbania** | 4576.05 ± 23.94 c | 21.88 ± 0.21 c | 21.45 ± 1.02 b | 45.15 ± 0.65 a |
| **Forage rapeseed** | 5007.21 ± 42.70 b | 47.20 ± 3.20 b | 18.37 ± 1.78 bc | 46.73 ± 3.30 a |
| **Forage oat** | 8743.17 ± 27.70 a | 24.45 ± 0.29 c | 25.83 ± 3.26 a | 47.36 ± 1.20 a |

Note: Different lowercase letters indicate significant differences among different green manure species (*P* < 0.05).
